# Mutation screening of germline *TP53* mutations in high-risk Chinese breast cancer patients

**DOI:** 10.1186/s12885-020-07476-y

**Published:** 2020-11-02

**Authors:** Ava Kwong, Vivian Yvonne Shin, Cecilia Y. S. Ho, Chun Hang Au, Thomas P. Slavin, Jeffrey N. Weitzel, Tsun-Leung Chan, Edmond S. K. Ma

**Affiliations:** 1 Department of Surgery, The University of Hong Kong and University of Hong Kong-Shenzhen Hospital, Hong Kong SAR, China; 2 Department of Surgery, Hong Kong Sanatorium & Hospital, Hong Kong SAR, China; 3 Hong Kong Hereditary Breast Cancer Family Registry, Hong Kong SAR, China; 4 Department of Pathology, Division of Molecular Pathology, Hong Kong Sanatorium & Hospital, Hong Kong SAR, China; 5 Department of Medical Oncology & Therapeutics Research, Division of Clinical Cancer Genetics, City of Hope, Duarte, CA USA; 6 Department of Population Sciences, Beckman Research Institute of City of Hope, Duarte, CA USA

**Keywords:** Hereditary breast cancer, *TP53* mutation, Chinese, Breast cancer risk

## Abstract

**Background:**

Germline *TP53* mutations are associated with Li-Fraumeni syndrome, a severe and rare hereditary cancer syndrome. Despite the rarity of germline *TP53* mutations, the clinical implication for mutation carriers and their families is significant. The risk management of *TP53* germline mutation carriers is more stringent than *BRCA* carriers, and radiotherapy should be avoided when possible.

**Methods:**

*TP53* gene mutation screening was performed in 2538 Chinese breast cancer patients who tested negative for *BRCA* mutations.

**Results:**

Twenty *TP53* mutations were identified with high next-generation sequencing concerning for germline mutations in Chinese breast cancer families. The majorities of the *TP53* carriers had early-onset, hormone receptor-positive breast cancer, and had strong family history of cancer. Among all, 11 patients carried a germline mutation and 6 of which were likely de novo germline mutations. In addition, 1 case was suspected to be induced by chemotherapy or radiation, as this patient had no significant family history of cancer and aberrant clonal expansion can commonly include *TP53* mutations. Furthermore, we have identified one mosaic LFS case. Two novel mutations (c.524_547dup and c.529_546del) were identified in patients with early-onset.

**Conclusions:**

In view of the high lifetime risk of malignancy, identification of patients with germline *TP53* mutations are important for clinicians to aid in accurate risk assessment and offer surveillance for patients and their families.

## Background

Li-Fraumeni syndrome (LFS) is a rare autosomal genetic disorder which is frequently associated with germline *TP53* mutations. Germline *TP53* mutations are seen in 70% of families with LFS features. Individual with the mutation commonly present with LFS spectrum tumors (sarcoma, brain tumor, adrenocortical carcinoma, leukemia, germ cell tumor and breast cancer) [1, 2]. The lifetime risk of breast cancer in *TP53* mutation carriers is up to 80–90%, which is even higher than those harboring a *BRCA1* or *BRCA2 (BRCA)* mutation: the most commonly identified high penetrance germline gene mutations in hereditary breast cancer [3].

Although rare, germline *TP53* mutations are estimated to occur in up to 1% of all breast cancer cases [4]. Very early-onset of breast cancer is a common characteristic of *TP53* mutation carriers in which the median age being 27–30 years old [5]. *TP53* breast tumors are usually enriched with HER2-positive receptors, and 84% are either estrogen and/or progesterone receptor positive [6, 7]. Patients with *TP53* mutations have also been shown to have a shorter survival when compared to non-carriers [8].

The National Comprehensive Cancer Network (NCCN) has published testing and management guidelines for *TP53* gene mutation carriers. Under the NCCN guidelines, *TP53* mutation testing is recommended for early-onset breast cancer patients (age of diagnosis < 31) or those who meet classic LFS or Chompret criteria. It is also recommended that women who are *TP53* mutations carriers have breast surveillance similar to that of *BRCA* mutation carriers, and in addition, receive an annual total body MRI scan and skin cancer screening, and colonoscopy every 2–5 years beginning at 25 years of age. Although there is still no actionable drug which can target *TP53* mutations with good clinical trial data support, evidence shows that patients who carried *TP53* mutation are less responsive to low dose radiation and have a higher risk of new malignancies induced by radiotherapy [9]. Hence, *TP53* mutation status does have its importance in clinical management.

To date, over 250 types of *TP53* mutations are included in the IARC *TP53* Database and over 70% of them are missense mutations (http://p53.iarc.fr/). Missense mutations are often more challenging to classify and interpret than loss of function mutations. Therefore, obtaining more phenotypic data on unique missense mutations is important for the literature. Furthermore, germline *TP53* literature has historically been confounded by aberrant clonal expansion (ACE) [10], an entity that describes low level hematopoietic progenitor cell mutations, usually in leukemia related genes confined to the blood compartment [11]. ACE has been shown to associate with advancing age and clinical exposures like chemotherapy and radiation. ACE can be a risk factor for leukemia, atherosclerotic vascular disease, and associated with increased all-cause mortality [11].

In a cohort of 240 Chinese women with early-onset of breast cancer (age < 35) or with first- or second-degree relatives with breast and/or ovarian cancer, *TP53* mutation carriers were found in 1% of the cohort [12]. However, in Canada, there were no pathogenic mutations identified in a cohort of 95 women with early-onset of breast cancer (age < 30) [13]. This suggests that the clinical characteristics of *TP53* carriers vary across different ethnicities and countries. The frequency of *TP53* mutations also remains largely unknown in the Chinese population, therefore, our study aims to investigate the prevalence of *TP53* mutation in Chinese breast cancer patients and unravel the clinical characteristics of *TP53* mutations in their families.

## Methods

### Participants and selection criteria

*TP53* gene mutation screening was performed on 2538 Chinese breast cancer patients with no *BRCA1* and *BRCA2* germline mutations. Patients were recruited from the Hong Kong Hereditary and High Risk Breast Cancer Program (HRBCP) through the Hong Kong Hereditary Breast Cancer Family Registry from March 2007 to August 2019. Patient selection criteria was as follows: (1) patients had at least one first- or second- degree relative with breast and/or ovarian cancer, regardless of age; (2) the age at breast cancer diagnosis was under 45 years; (3) patients with bilateral breast cancer; (4) patients with triple-negative hormone receptors breast cancers, (5) cancers with medullary type histology; (6) patients having at least one relative with *BRCA-*associated cancer other than breast and ovarian cancer (such as stomach or prostate cancers) or known to be *BRCA* mutation related family; (7) patients with male breast cancer.

### DNA extraction

Blood, hair follicles and/or buccal swab DNA samples were collected from patients. Genomic DNA extraction was performed using QIAamp DNA Blood Mini Kit (Qiagen, Hilden, Germany) or QIAsymphony DNA Mini Kit (Qiagen) according to manufacturer’s instructions. Genomic DNA was quantified using a Qubit dsDNA BR Assay Kit and a Qubit 2.0 Fluorometer (Life Technologies, USA).

### Sequencing of *TP53* gene

Extracted DNA was applied to the QIAseq Human *BRCA1* and *BRCA2* Plus Panel DHS-103Z (Qiagen). Sequencing libraries were prepared according the QIAseq™ Targeted DNA Panel protocol (Qiagen). The libraries were pooled and sequenced on MiSeq or NextSeq (Illumina, San Diego, CA) instruments to reach minimum sequencing depth of 50-fold. Median coverage typically ranged between 200-300X.

To confirm germline mutations, Sanger sequencing of specific mutations was carried out on blood, hair follicles, and/or buccal swab DNA.

### Bioinformatics analysis

The bioinformatics analysis was performed on a Cray XC30 supercomputer (Cray, Seattle, WA). Paired sequencing reads were mapped to the human reference genome sequence GRCh37/hg19 using BWA-MEM v0.7.7 by default parameters [14]. Post-alignment primer clipping and unique molecular identifier (UMI) extraction were performed using BAMClipper [15]. Samples having at least 75% gene-specific primers with at least 100 detected UMI per primer were considered to pass quality control and subject to variant calling by FreeBayes v1.0.2–15 [16]. Called variants with variant allelic fraction (VAF) of at least 5% were annotated by Ensembl Variant Effect Predictor v75 [17]. Variants with minor allele frequency of at least 1% reported by The 1000 Genomes Projects [18] were excluded from manual variant curation. Variants in exon and at least 10 bp of the flanking introns were reported and described according to the standardized recommendations of the Human Genome Variation Society (HGVS) nomenclature [19]. Variant descriptions were checked by IARC TP53 database (http://p53.iarc.fr/) and Mutalyzer Name Checker (http://mutalyzer.nl). Variants in this study were interpreted based on classification from ClinVar database (https://www.ncbi.nlm.nih.gov/clinvar/) with clinical adjustment with reference to the classic for Li-Fraumeni syndrome criteria [2].

### Molecular analysis of de novo germline mutations

Mutations from families in which both parents tested negative were presumed as de novo mutations. Haplotype analysis was also performed to confirm de novo cases. In cases where the patient had no first or second-degree relatives with cancer history or positive test result, and the blood samples from the patient’s parent were unavailable, the patient was considered as likely de novo.

### Statistical analysis

Fisher’s exact test and Wilcoxon rank sum test were used to study the relationship between clinicopathological variables and mutation status. The limit of significance for all analyses was defined as *P*-value of < 0.05. Data analyses were performed using statistical software R (version 3.4.2) [20].

## Results

In a cohort of 2538 breast cancer patients, there were 28 *PALB2* and 2 *PTEN* mutation identified, which were excluded from the study. Among 2508 patients, the mean age at diagnosis was 45.63 years (range 18–95). Of all primary tumors, 1760 (75.41%) were hormone receptor positive, 211 (9.04%) were HER2+, and 327 (14.01%) were triple-negative. A positive family history of breast cancer (first- or second-degree relatives) was seen among 922 (36.76%) of the patients and 473 (18.86%) of the patients had a family history with ≥3 different types of cancers in their first- and second-degree relatives.

*TP53* mutations were infrequent in this cohort. Only 20 different mutations (0.80%) were identified among the 2508 breast cancer patients. The mean age at diagnosis of breast cancer for the mutation carriers and non-carriers were 31.65 years and 45.74 years (*p*-value < 0.001), respectively. In *TP53* mutation carriers, the majority of the tumors were hormone receptor-positive (16/21, 76.19%) (OR compare with non-carriers: 1.04, 95% CI: 0.363–3.661; *p*-value =1). A positive family history of breast cancer (among first- and second- degree relatives) was reported in 5 (25%) *TP53* mutation carriers compared to 917 (36.86%) non-carriers (OR 0.571, 95% CI: 0.162–1.660, *p*-value = 0.355). Moreover, there were 8 (40%) mutation carriers with a family history of ≥3 different types of cancers in first- and second-degree relatives compared to 465 (18.69%) of non-carriers (OR: 2.90, 95% CI 1.022–7.764, p-value = 0.038). Characteristics of mutation carriers and non-carriers are shown in Table 1.

**Table 1:** Characteristics of Chinese breast cancer patients screened for *TP53* mutations

	Mutation Negative	%	***TP53+***	%	Total	%	P-value
	***N*** = 2488		***N*** = 20		***N*** = 2508		(Wilcoxon rank sum test/Fisher Exact Test)
**Mean/Median age at Diagnosis**	45.74/44		31.65/30		45.63/44		< 0.001
**Age range**	18–95		18–47		18–95		
**Bilateral cases**	439	17.64%	12	60.00%	451	17.98%	< 0.001
**Age at breast cancer diagnosis**							
**≤ 29**	116	4.66%	9	45.00%	125	4.98%	< 0.001
**30–39**	637	25.60%	6	30.00%	643	25.64%	
**40–49**	998	40.11%	5	25.00%	1003	39.99%	
**≥ 50**	737	29.62%	0	0.00%	737	29.39%	
**Family history of breast cancer** (in first and second degree relatives)							
**Yes**	917	36.86%	5	25.00%	922	36.76%	0.355
**No**	1571	63.14%	15	75.00%	1586	63.24%	
**Family history of > =3 different types of cancers** (in first and second degree relatives)							
**Yes**	465	18.69%	8	40.00%	473	18.86%	0.038
**No**	2023	81.31%	12	60.00%	2035	81.14%	
**Histology**^**a**^							
	*N* = 2927		*N* = 32		*N* = 2959		
**Ductal**	1991	71.21%	17	58.62%	2008	71.08%	0.283
**Lobular**	94	3.36%	1	3.45%	95	3.36%	
**DCIS**	498	17.81%	9	31.03%	507	17.95%	
**Others**	213	7.62%	2	6.90%	215	7.61%	
**Unclassified**	131	–	3	–	134	–	
**Molecular subtypes**^**a**^ **(excluded in-situ CA)**							
	*N* = 2429		*N* = 23		*N* = 2452		
**Hormone receptor +**	1744	75.40%	16	76.19%	1760	75.41%	0.291
**Hormone receptor -**	36	1.56%	0	0.00%	36	1.54%	
**HER2+**	207	8.95%	4	19.05%	211	9.04%	
**TNBC**	326	14.09%	1	4.76%	327	14.01%	
**Unclassified**	116	–	2	–	118	–	

*Abbreviation*: *DCIS* ductal carcinoma in situ, *HER2* human epidermal growth factor receptor 2, *TNBC* Triple-negative breast cancer

^**a**^Count for each primary of bilateral cases

**Fig. 1:**
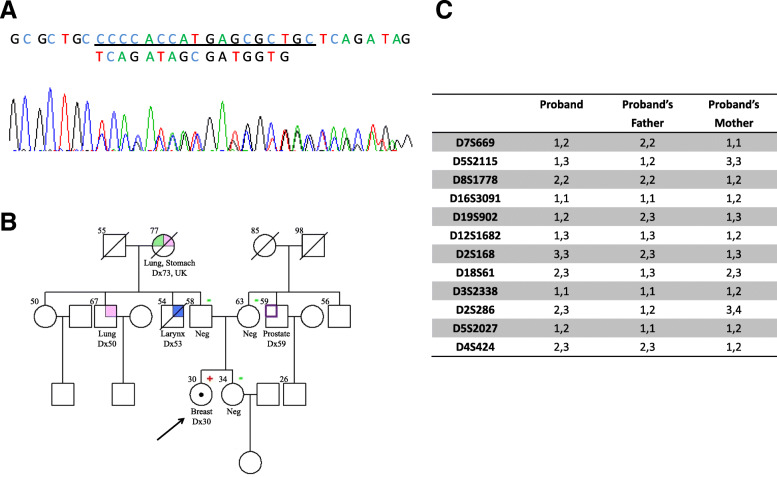
De novo mutation of *TP53*. **a** Sanger sequencing of codon 526–562 of wide type and c.529_546del mutant of *TP53* gene. **b** The pedigree of family F06 with information including age, tumor type and tumor onset age. “**+**” = affected subject; “**-**” unaffected subject. **c** Haplotype analysis for family F06

The majority of the mutations (15/20) identified were missense mutations, followed by 2 nonsense mutations, 2 deletions/insertions and 1 splice site mutation (Tables 2 and 3). By testing ancillary materials, multiple germ layers and/or clinical data to interrogate germline status on the 20 carriers, 11 (55%) patients were confirmed to carry a germline mutation, 2 (10%) patients were confirmed to have de novo germline mutations (Fig. 1), and 4 (20%) were presumed to have de novo germline mutations based on the negative test result of *TP53* mutation among multiple family members and/or lack of cancer history in families. Five of the patients were deceased and three of the patients refused further investigation on family studies, some information were no longer traceable. In all, 70% had early-onset of breast cancer (< 35 years) and 60% had bilateral breast cancer. Interestingly, we found that 25% (5/20) of the patients had no family history of cancer, 2 patients had bilateral breast cancer, one had bilateral breast cancer and thyroid cancer and one had multiple cancers in breast and brain.

**Table 2:** Characteristics of Chinese breast CA patients (no chemo/radiotherapy received before genetic test) identified with germline *TP53* mutation

Patient ID	Nucleotide alteration	Clin-var	Number of cases in IARC TP53 (Asian/All)	VAF (%)	Germline	Germline evidence(s)				C/R	Tumor types	Age at Dx	Mutation type	Stage of disease	Vital status	Family history	LFS/ LFL	Classifi-cation
						FM	HF	BS	PT									
F01	c.96 + 1G > T; Del of exon3 r.75_96del p.Leu26Profs*11	P/LP	0/0	51.5	Confirmed germline	–	+	+	NA	Y	Breast CA Breast CA	18 43	Splice-Site Mutation	Stage I Stage 0	Alive	Breast CA, Colorectal CA, Brain and CNS Tumors	LFL	Chompret
F02	c.422G > A; p.Cys141Tyr	P	5/11	56.9	Confirmed Germline; DeNovo	–	+	+	Y	N	Breast CA, Breast CA, Thyroid CA	30 34 32	Missense Mutation	Stage II Stage 0	Alive	Colorectal CA, Lung CA	LFL	Chompret
F03	c.473G > A; p.Arg158His	P/LP	2/23	51.4	Suspected germline^b^	–	NA	NA	NA	Y	Breast CA	47	Missense Mutation	Stage III	Deceased	Multiple Lung CAs, Colorectal CA, Brain and CNS Tumor	LFL	N
F04	c.490A > G; p.Lys164Glu	VUS	0/0	54.3	Confirmed germline	+	/	/	/	N	Breast CA	24	Missense Mutation	Stage 0	Alive	Osteosarcoma, Soft Tissue Tumor, NPC	LFS	Chompret
F05	c.527G > T; p.Cys176Phe	LP	0/1	36.9	Suspected germline^b^	NA	NA	NA	NA	Y	Breast CA, Breast CA Ovarian CA Lung CA	22 31 38 38	Missense Mutation	Stage II Stage 0	Alive	Liver CA, Stomach CA	N	Chompret
F06	c.529_546del; p.Pro177Cys182del	NI	Novel	33.1	Confirmed Germline; DeNovo	–	+	+	Y	N	Breast CA	30	Missense Mutation	Stage II	Alive	Multiple Lung CAs, Stomach CA, Laryngeal CA Prostate CA	LFL	Chompret
F07	c.536A > G; p.His179Arg	VUS	2/2	48.5	Confirmed germline	+	/	/	/	Y	Breast CA, Breast CA	33 33	Missense Mutation	Stage II Stage I	Deceased	Esophageal CAs, Stomach CA, Lung CA, Stomach CA, Bone CA, Breast CA, Connective Tissue CA	LFS	Chompret
F08	c.541C > T; Arg181Cys	P/LP/ VUS	17/21	55.1	Suspected germline^b^	NA	NA	NA	NA	Y	Breast CA, Breast CA	36 42	Missense Mutation	Stage II Stage I	Alive	Lung CA, Testicular CA Ovarian CA	LFL	N
F09	c.626_627dupGA; p.Asn210Glufs*38	NI	0/3	35.0	Confirmed Germline; Likely DeNovo	–	+	+	NA	N	Breast CA, Breast CA Thyroid CA	30 46 53	Insertion	Stage 0Stage 0	Alive	/	N	Chompret
F10	c.638G > A; p.Arg213Gln	P	5/16	-^a^	Confirmed germline	+	/	/	/	Y	Breast CA, Thymus CA, Adrenocortical CA	28 25 5	Missense Mutation	Stage II	Alive	Thyroid CA, Lung CA, Brain and CNS Tumors	LFL	Chompret
F11	c.722C > T; p.Ser241Phe	LP	3/6	65.8	Suspected germline^b^	–	NA	NA	NA	Y	Breast CA, Breast CA	25 32	Missense Mutation	Stage 0 Stage I	Deceased	Colorectal CA, Breast CA	LFL	Chompret
F12	c.743G > A; p.Arg248Gln	P/LP	14/64	50.9	Confirmed germline	+	/	/	/	Y	Breast CA, Breast CA	44 44	Missense Mutation	Stage IIA Stage IIA	Alive	Sarcoma Multiple Breast CAs Stomach CA	LFS	Chompret
F13	c.818G > A; p.Arg273His	P/LP	23/75	48.3	Confirmed germline	NA	+	+	NA	N	Breast CA, Breast CA	28 30	Missense Mutation	Stage 0Stage 0	Alive	Unknown CA	N	Chompret
F14	c.825 T > G; p.Cys275Trp	LP	0/0	43.7	Germline; Likely DeNovo	–	NA	NA	NA	N	Breast CA, Breast CA	28 35	Missense Mutation	Stage I unknown	Deceased	/	N	Chompret
F15	c.844C > T; p.Arg282Trp	P/LP	13/52	51.7	Germline; Likely DeNovo	NA	NA	NA	NA	N	Breast CA, Brain and CNS Tumors	27 30	Missense Mutation	Stage II	Deceased	/	N	Chompret
F16	c.916C > T; p.Arg306*	P	9/33	44.3	Confirmed Germline; Likely DeNovo	–	+	+	NA	N	Breast CA	32	Nonsense Mutation	Stage I	Alive	Lung CA Oral CA Colorectal CA	LSL	Chompret
F17	c.1010G > A; p.Arg337His	P	2/112	42.4	Confirmed germline	+	/	/	/	Y	Breast CA, Lung CA	42 54	Missense Mutation	Stage II	Alive	Colorectal CA, Uterus CA, Multiple Breast CAs, Multiple Lung CAs	LFS	Chompret
F18	c.1025G > C; p.Arg342Pro	P/LP	2/6	51.0	Suspected germline^b^	NA	NA	NA	NA	N	Breast CA, Breast CA	23 23	Missense Mutation	Stage I Stage II	Alive	Nasopharyngeal CA, Liver CA	N	Chompret

LFS (Classic Li-Fraumeni syndrome): an individual diagnosed age < 45 with a sarcoma AND a first degree relative diagnosed age < 45 with cancer AND an additional first or second degree relative in the same linage with cancer diagnosed age < 45 or with a sarcoma at any age [1]

LFL (Li-Fraumeni-like Syndrome): an individual with two first- or second degree relatives with LFS tumors spectrum at any age, rather than the three required by the classic criteria [20] OR proband with any childhood cancer or sarcoma, brain tumor, or adrenocortical carcinoma diagnosed under 45 years of age, with one first or second degree relative with typical LFS cancer diagnosed at any age, plus one first or second degree relative in the same lineage with any cancer diagnosed under age 60 [19]

Chompret: Individual with a tumor from LFS tumor spectrum diagnosed < 46 AND at least one first or second degree relative with any of the aforementioned cancers (other than breast cancer if the proband has breast cancer < 56 OR individual with multiple primaries at any age OR individual with multiple tumors (excluding multiple breast tumors), two of which belong to LFS tumors spectrum with the initial cancer occurring < 46 OR individual with adrenocortical carcinoma or choroid plexus carcinoma or rhabdomyosarcoma of embryonal anaplastic subtype at any age, regardless of family history or breast cancer diagnosed < 31 [21].

*Abbreviations*: *FM* family member(s); HF: Hair follicle; *BS* buccal swab; *PT* Paternity test; *C/R* Chemotherapy or radiotherapy; *NI* No information; *NA* Not available; *Dx* Diagnosis; *VAF* variant allele frequency; *P* Pathogenic; *LP* Likely pathogenic; *VUS* Variant of unknown significance

^a^Known *TP53* Family

^b^Suspected germline: an individual who meet either LFS or LFL or Chompret criteria but family study or other germline layer(s) were not available

**Table 3:** Characteristics of Chinese breast CA patients identified with aberrant clonal expansion *TP53* mutation or mosaic *TP53* mutation

Patient ID	Nucleotide alteration	Clin-var	Number of cases in IARC (Asian/All)	VAF (%)	Germline (deNovo)/ACE	Germline evidence(s)				C/R	Tumor types	Age at Dx	Mutation type	Stage of disease	Vital status	Family history	LFS/LFL	Classification
						FM	HF	BS	PT									
F19	c.524_547dup; p.Cys182_Ser183insCys CysProHisHisGluArgCys	NI	Novel	17.5	Mosaic	–	L	L	NA	N	Breast CA	40	Insertion	Stage II	Alive	/	N	N
F20	c.775G > A; p.Asp259Asn	NI	Novel	58.7	Likely ACE^a^	NA	NA	NA	NA	Y	BreastCA, Breast CA	46 48	Missense Mutation	Stage II Stage I	Alive	/	N	N

LFS (Classic Li-Fraumeni syndrome): an individual diagnosed age < 45 with a sarcoma AND a first degree relative diagnosed age < 45 with cancer AND an additional first or second degree relative in the same linage with cancer diagnosed age < 45 or with a sarcoma at any age [1]

LFL (Li-Fraumeni-like Syndrome): an individual with two first- or second degree relatives with LFS tumors spectrum at any age, rather than the three required by the classic criteria [20] OR proband with any childhood cancer or sarcoma, brain tumor, or adrenocortical carcinoma diagnosed under 45 years of age, with one first or second degree relative with typical LFS cancer diagnosed at any age, plus one first or second degree relative in the same lineage with any cancer diagnosed under age 60 [19]

Chompret: Individual with a tumor from LFS tumor spectrum diagnosed < 46 AND at least one first or second degree relative with any of the aforementioned cancers (other than breast cancer if the proband has breast cancer < 56 OR individual with multiple primaries at any age OR individual with multiple tumors (excluding multiple breast tumors), two of which belong to LFS tumors spectrum with the initial cancer occurring < 46 OR individual with adrenocortical carcinoma or choroid plexus carcinoma or rhabdomyosarcoma of embryonal anaplastic subtype at any age, regardless of family history or breast cancer diagnosed < 31 [21].

*Abbreviations*: *FM* family member(s); *HF* hair follicle; *BS* buccal swab; *PT* paternity test; *C/R* chemotherapy or radiotherapy; *NI* no information; *NA* not available; *Dx* diagnosis; *VAF* variant allele frequency; *ACE* aberrant clonal expansion; *P* pathogenic; *LP* likely pathogenic; *VUS* variant of unknown significance; *L* low VAF

^a^Likely ACE: an individual diagnosed age < 35 AND with no family history of cancer(s) AND received chemotherapy or radiotherapy before genetic test

Novel mutations (c.524_547dup and c.529_546del) were seen in 2 patients with both diagnosed breast cancer at age below 40. One of the novel mutation carriers (F19) shows equivocal result in both of her blood, hair follicle and buccal swab DNA with trace amount of *TP53* duplication, serving as evidence of mosaicism or ACE (in Table 3). The other novel *TP53* carrier (F06) was de novo germline mutation, with significance family history of cancer.

In this cohort, there were 5 suspected germline mutation cases (F03, F05, F08, F11 and F18); these patients had strong family history with cancers and fulfil either Li-Fraumeni-like (LFL) criteria and/or Chompret criteria, however, family cascade testing was not possible due to loss of follow-up or family members refusing testing. In addition, we suspected that one of the cases (F20) was likely ACE induced by chemotherapy or radiation (Table 3); this patient had breast cancers at age > 45, received chemotherapy before the genetic test, and had no other significant personal or family history of cancer and hence is likely not a germline related.

## Discussion

Among 2508 Chinese breast cancer patients, we identified 18 germline *TP53* and 2 ACE/mosaic *TP53* cases. Of 18 germline cases, two of them did not meet the NCCN guidelines for *TP53* genetic testing but the families had LFL syndrome (Table 2). In general, germline *TP53* families had at least one member with LFS tumor spectrum i.e. sarcoma, brain tumor, breast cancer, leukemia, bronchoalveolar lung carcinomas, germ cell tumor or adrenocortical carcinoma [1, 2, 21–23]. However, we found that 16.67% (3 of 18) of the patients had no family history of cancer.

Among 374 patients in our study with early-onset breast cancer (age < 35), the detection rate of a *TP53* germline mutation was 3.74% which is comparable to other studies in the West (2–7.1%) [24, 25], and among Chinese high risk breast patients (1–5%) [12, 26, 27] (Table 2). A study of French-Canadian cancer families suggested that women with breast cancer before the age of 50 with no family history of cancer still warrant screening for *TP53* mutations, even though the mutation frequency (0.5%) is low compare to *BRCA* mutations (4.8%) [28].

Interestingly, there were 6 (33.3%) de novo or likely de novo cases. Another study on early-onset cancer study suggests that the frequency of de novo *TP53* mutations is 7–20% [29]. There were two *TP53* mutations, c.490A > G (F04) and c.536A > G (F07) both of the families showed characteristics of classical LFS. Their families have significant family history of sarcoma, although ClinVar has classified them as variant of unknown significance (VUS), we believe that the pathogenicity of these two variants should be further determined based on their family histories. With a significant family history of cancers, these families have been offered high risk surveillance.

The mutation *TP53,* c.1010G > A, has been previously reported as founder mutation in Southern Brazilian [30]. Interestingly, it was detected in one of the Chinese families who had breast and lung cancer and multiple family cancers. The mutation *TP53*, c.529_546del, has been identified somatically in thyroid cancer [31], small cell lung cancer [32] and breast cancer [24, 33]. We detected this mutation in one of the families who had breast cancer at age 30 and a family history of multiple cancers. The variant allele fraction (VAF) was at 33% by NGS, which was lower than the average range of *TP53* VAFs identified in our study. Further analysis was performed on a buccal swab by Sanger sequencing in which the VAF was ~ 50% and therefore the mutation was confirmed to be germline.

In another family (F19), we detected a 17.5% VAF by NGS, which was much lower than the normal germline range of 40–60% VAF. Further analysis on hair follicles and buccal swab by both NGS and Sanger sequencing showed trace amounts of the mutation, therefore testing on tumor tissue would be able to confirm somatic mosaicism or ACE [10], however, the patient received neoadjuvant chemotherapy before surgery and there was no tissue available for further testing.

Radiation induced genomic instability causing aberrant hematopoietic stem/progenitor cells mobilized into the peripheral blood circulation result in ACE sometimes involving *TP53* [10, 34]. In one of the patients who was only tested after chemotherapy has been administered, breast cancer was diagnosed at old age (> 35) and there was no cancer history in their families, suggesting the variant was more likely due to ACE rather than LFS.

Increased risk of secondary malignancies in *TP53* mutation carriers with radiation exposure has been reported [35]. In a preclinical study of 6 germline *TP53* mutated breast cancer patients who received adjuvant radiotherapy, 3 later developed ipsilateral breast recurrences, 4 developed contralateral breast cancers, 2 developed radiotherapy-induced cancers, and 2 developed new primaries (1 of which was an ipsilateral chest wall angiosarcoma and the other was a grade 2 ethmoidal leiomyosarcoma) [36].

## Conclusion

Overall, our study shows the spectrum of *TP53* germline mutations in a Chinese cohort and also clinical characteristics of Chinese *TP53* carriers and their families which may help clinicians identify patients for *TP53* mutation screening. Young aged (even without a cancer family history) women with breast cancer is a major association and should be considered for *TP53* genetic testing. Identification of a *TP53* mutation may also affect the treatment options for these patients, i.e. potentially minimizing the use of radiation to prevent radiation-related malignancies [37]. Moreover, our findings may aid in the development of new guidelines for *TP53* screening in breast cancer patients with Chinese ethnicity.

## Supplementary information

**Additional file 1.**

## Data Availability

The data that support the findings of this study are available from the corresponding author upon reasonable request.

## Abbreviations

ACE: Aberrant clonal expansion
HRBCP: Hong Kong Hereditary and High Risk Breast Cancer Program
HGVS: Human Genome Variation Society
LFS: Li-Fraumeni syndrome
NCCN: The National Comprehensive Cancer Network
UMI: Unique molecular identifier
VAF: Variant allelic fraction
