# Supplementary material for: Mutation screening of germline TP53 mutations in high-risk Chinese breast cancer patients
Source: BMC Cancer. 2020 Nov 2;20:1053. doi: 10.1186/s12885-020-07476-y (PMC7607817; doi:10.1186/s12885-020-07476-y)
Supplement: Supplementary file 1 — Additional file 1. [file 12885_2020_7476_MOESM1_ESM.docx]

Supplementary 1

Human Breast Cancer Panel from QIAseq Targeted DNA DHS-001Z Panel (Qiagen, Hilden, Germany) detects mutations in 93 breast cancer predisposition genes.

**Carcinoma**

Adenoid Cystic Carcinoma: *PIK3CA (p110-alpha)*, *PTEN*.

Basal (Triple-Negative) Carcinoma: *ATR*, *BLM*, *BRCA1*, *BRCA2*, *CASP8 (FLICE)*, *CDH1 (E-Cadherin)*, *CDKN2A (P16INK4A)*, *CSMD1*, *EGFR (ERBB1)*, *ERBB2 (HER-2, NEU)*, *ERBB3*, *EXOC2*, *FGFR1*, *HERC1*, *ITCH*, *KMT2C*, *KRAS*, *MED12*, *MSH6*, *MUC16*, *NEK2*, *PALLD*, *PIK3CA (p110-alpha)*, *PIK3R1 (p85-ALPHA)*, *PMS2*, *PTEN*, *PTGFR*, *RAD51C*, *RB1*, *SMARCA4*, *SYNE1*, *TP53 (p53)*, *XRCC2*.

Ductal Carcinoma: *AKT1*, *APC*, *ATM*, *ATR*, *BRCA1*, *BRCA2*, *CDH1 (E-Cadherin)*, *CDKN2A (P16INK4A)*, *CSMD1*, *EGFR (ERBB1)*, *ERBB2 (HER-2, NEU)*, *FANCC*, *FGFR2*, *GATA3*, *GEN1*, *HERC1*, *HOXB13*, *KMT2C*, *KRAS*, *MED12*, *MLH1*, *MRE11*, *MSH2*, *MUC16*, *NF1*, *PALB2*, *PALLD*, *PIK3CA (p110-alpha)*, *PIK3R1 (p85-ALPHA)*, *PTEN*, *RAD51D*, *SMAD4 (MADH4)*, *STK11 (LKB1)*, *SYNE1*, *TP53 (p53)*.

Ductolobular Carcinoma: *PIK3CA (p110-alpha)*.

ER-Positive & PR-Positive Carcinoma: *ACVR1B*, *AKT1*, *APC*, *CBFB*, *CDH1 (E-Cadherin)*, *ERBB3*, *EXT2*, *HERC1*, *KRAS*, *MLH1*, *MRE11*, *PIK3CA (p110-alpha)*, *PPM1L*, *PTEN*, *SEPT9*, *TP53 (p53)*.

HER-Positive Carcinoma: *APC*, *AR*, *BMPR1A (ALK3)*, *CASP8 (FLICE)*, *CDH1 (E-Cadherin)*, *CDK4*, *FBXO32*, *HERC1*, *IRAK4*, *ITCH*, *KMT2C*, *MLH1*, *MSH6*, *MUC16*, *NCOR1*, *NF1*, *PALB2*, *PIK3CA (p110-alpha)*, *PTGFR*, *RAD50*, *SMAD4 (MADH4)*, *STK11 (LKB1)*, *TP53 (p53)*, *TRAF5*.

Lobular Carcinoma: *AKT1*, *ATR*, *CDH1 (E-Cadherin)*, *ERBB2 (HER-2, NEU)*, *MSH2*, *NF1*, *PALB2*, *PIK3CA (p110-alpha)*, *RB1*, *TP53 (p53)*, *VHL*.

Luminal Carcinoma: *APC*, *CDH1 (E-Cadherin)*, *CDKN2A (P16INK4A)*, *CSMD1*, *GATA3*, *KRAS*, *MAP2K4 (MKK4, JNKK1)*, *NBN*, *PIK3CA (p110-alpha)*, *PTEN*, *TP53 (p53)*.

Other Carcinomas: *APC*, *AR*, *ATR*, *AXIN2*, *BARD1*, *BRIP1*, *CASP8 (FLICE)*, *CDK6*, *CHEK2 (RAD53)*, *CSMD1*, *CTNNB1*, *DIRAS3*, *ERCC4*, *FAM175A*, *FANCC*, *GEN1*, *HERC1*, *HOXB13*, *KRAS*, *MED12*, *MEN1*, *MRE11*, *MSH6*, *MUTYH*, *NBN*, *NF1*, *PALB2*, *PALLD*, *PMS1*, *PMS2*, *RAD50*, *RAD51*, *RAD51C*, *SMAD4 (MADH4)*, *SMARCA4*, *STK11 (LKB1)*, *SYNE1*, *TGFB1*.

Other Carcinoma-Related Genes: *BAP1*, *EP300*, *ESR1 (ERα)*, *MAP3K1 (MEKK1)*, *MDM2*, *MYC*, *PBRM1*, *PCGF2*, *WEE1*, *ZBED4*.

**Carcinoma in situ**

Ductal & Lobular Carcinoma in situ: *CDH1* *(E-Cadherin)*.

Ductal Carcinoma in situ: *AKT1*, *CDH1 (E-Cadherin)*, *PIK3CA (p110-alpha)*, *TP53 (p53)*.

Lobular Carcinoma in situ: *CDH1 (E-Cadherin)*.
